# The Flowering Repressor *SVP* Underlies a Novel *Arabidopsis thaliana* QTL Interacting with the Genetic Background

**DOI:** 10.1371/journal.pgen.1003289

**Published:** 2013-01-31

**Authors:** Belén Méndez-Vigo, José M. Martínez-Zapater, Carlos Alonso-Blanco

**Affiliations:** 1 Departamento de Genética Molecular de Plantas, Centro Nacional de Biotecnología (CNB), Consejo Superior de Investigaciones Científicas (CSIC), Madrid, Spain; 2 Instituto de Ciencias de la Vid y del Vino (ICVV), Consejo Superior de Investigaciones Científicas (CSIC), Universidad de La Rioja, Gobierno de La Rioja, Logroño, Spain; University of California Davis, United States of America

## Abstract

The timing of flowering initiation is a fundamental trait for the adaptation of annual plants to different environments. Large amounts of intraspecific quantitative variation have been described for it among natural accessions of many species, but the molecular and evolutionary mechanisms underlying this genetic variation are mainly being determined in the model plant *Arabidopsis thaliana*. To find novel *A. thaliana* flowering QTL, we developed introgression lines from the Japanese accession Fuk, which was selected based on the substantial transgression observed in an F_2_ population with the reference strain L*er*. Analysis of an early flowering line carrying a single Fuk introgression identified *Flowering Arabidopsis QTL1* (*FAQ1*). We fine-mapped *FAQ1* in an 11 kb genomic region containing the MADS transcription factor gene *SHORT VEGETATIVE PHASE* (*SVP*). Complementation of the early flowering phenotype of *FAQ1*-Fuk with a *SVP*-L*er* transgen demonstrated that *FAQ1* is *SVP*. We further proved by directed mutagenesis and transgenesis that a single amino acid substitution in *SVP* causes the loss-of-function and early flowering of Fuk allele. Analysis of a worldwide collection of accessions detected *FAQ1/SVP*-Fuk allele only in Asia, with the highest frequency appearing in Japan, where we could also detect a potential ancestral genotype of *FAQ1/SVP*-Fuk. In addition, we evaluated allelic and epistatic interactions of *SVP* natural alleles by analysing more than one hundred transgenic lines carrying L*er* or Fuk *SVP* alleles in five genetic backgrounds. Quantitative analyses of these lines showed that *FAQ1/SVP* effects vary from large to small depending on the genetic background. These results support that the flowering repressor *SVP* has been recently selected in *A. thaliana* as a target for early flowering, and evidence the relevance of genetic interactions for the intraspecific evolution of *FAQ1/SVP* and flowering time.

## Author Summary

In many plant species, the timing of flowering initiation shows abundant quantitative variation among natural varieties, which reflects the importance of this trait for adaptation to different environments. Currently, a major goal in plant biology is to determine the molecular and evolutionary bases of this natural genetic variation. In this study we demonstrate that the central flowering regulator *SHORT VEGETATIVE PHASE* (*SVP*), encoding a MADS transcription factor, is involved in the flowering natural variation of the model organism *Arabidopsis thaliana*. In particular, we prove that a structural change caused by a single amino acid substitution generates a *SVP* early flowering allele that is distributed only in Asia. Furthermore, genetic interactions have been shown to be a component of the natural variation for many important adaptive traits. However, very few studies, either in animals or plants, have systematically addressed the extent of genetic interactions among specific alleles responsible for the natural variation of complex traits. Our study shows that the flowering effects of *SVP* natural alleles depend significantly on the genetic background; and, subsequently, we demonstrate the relevance of epistasis for the evolution of this crucial transcription factor and flowering time.

## Introduction

Flowering initiation is an essential developmental transition in plant life because it determines the timing of sexual reproduction. This transition is regulated by different environmental signals that synchronize reproduction with the most favourable season for seed production. Hence, the timing of flowering is a crucial adaptive trait in annual plants, since it will affect their survival and reproductive yield [1]. Supporting this relevance, considerable intraspecific quantitative variation has been classically described for flowering time among natural accessions or crop varieties for many annuals, which is presumed to reflect adaptation to local environments [2], [3]. In the past fifteen years there has been an unprecedented advance in our understanding of the molecular mechanisms of flowering regulation, mostly achieved by genetic studies of artificially induced mutants in the model plant *Arabidopsis thaliana* [4]. More than 100 flowering genes have been identified whose analyses are defining a complex regulatory network that involves several flowering pathways integrating different environmental signals. This network includes, among others, the photoperiod, the vernalization and the autonomous pathways, as well as various regulatory genes that play a role as pathway integrators, such as *FT* and *SOC1* [5]–[7]. Presently, a major aim in plant biology is to decipher the molecular and evolutionary bases of the naturally-existing genetic variation, for which *A. thaliana* has also become a promising model species [1], [8]–[10].

*A. thaliana* is broadly distributed as a native species in Eurasia, whereas it has been later introduced in North America and Japan, as well as in Australia and South America (reviewed in [11]). The large amount of natural genetic variation that has been described for flowering time is likely involved in adaptation to the contrasting climates that are covered by *A. thaliana* geographic distribution because this variation has been associated with latitude, altitude and climatic factors [12]–[16]. *A. thaliana* accessions have been qualitatively classified for long time as winter- or summer-annuals depending on their extreme late or early flowering behaviours and their high or low response to vernalization, respectively [17]. Mendelian genetic analyses identified two flowering repressors, *FRI* and *FLC*, as major determinants of such qualitative flowering differences [18], [19]. In addition, numerous quantitative trait locus (QTL) analyses have been carried out with different sorts of experimental mapping populations including F_2_ families [20], recombinant inbred lines (RILs) [21]–[27], introgression lines (ILs) [28], [29], advanced multiparent populations [30], [31], or collections of accessions [32], [33] grown in distinct environments. Each population detected between two and 13 QTL, which together correspond to, at least, 20 different genomic regions [9], [20]. Overall, these studies identified a few large effect QTL per population and a similar or higher number of small effect loci, thus showing the contribution of both extreme kinds of loci to the quantitative flowering time variation. Furthermore, despite the limitations to find genetic interactions among QTL (epistasis), owing to the low-order (two-way) level and small population sizes that can be tested, several analyses have detected a considerable number of significant interactions [20], [24], [25], [31], which indicates that epistasis is also an important genetic component of flowering time variation [34]. Even so, until now, only the well documented genetic interactions between *FRI* and *FLC* have been confirmed at the level of specific natural flowering alleles and described in terms of genetic networks [9], [35], [36]. Understanding the functional bases of genetic interactions among the specific alleles responsible for the natural variation of complex traits goes nowadays beyond the classical distinction between Fisher's and Wright's models of evolution [37] because epistasis lies below the networks currently pursued by system biology approaches [38], [39]. Therefore, functional studies addressing epistasis among natural alleles are required to determine its extent on flowering time variation and its consequences on the estimates of flowering QTL effects.

As a first step to understand the molecular mechanisms accounting for the natural quantitative variation for flowering time, multiple laboratories are pursuing the isolation of genes underlying *A. thaliana* QTL and the identification of nucleotide polymorphisms affecting the function of those genes. By using combinations of different functional approaches, twelve genes have been identified as large effect flowering QTL. These include the photoreceptor genes *CRY2*, *PhyC* and *PhyD*; the MADS transcription factor genes *FLC*, *FLM* and *MAF2*; *FRIGIDA* (*FRI*) and the *FRI*-like genes, *FRL1* and *FRL2*, encoding homologous proteins with unknown cellular function; the RNA processing gene *HUA2*; the circadian rhythm gene *ELF3*, and the florigen encoding gene *FT* (reviewed in [9], [10] and [24], [40], [41]. Detailed analyses of these genes have found indels or premature stop codons causing loss-of-function alleles, as well as amino acid substitutions and other structural modifications leading to functional changes [9], [40], [41]. In addition, several *cis*-regulatory polymorphisms have been demonstrated to alter gene expression levels [42], [43]. Interestingly, numerous series of independent loss-of-function alleles have been described for *FRI* and *FLC* [15], [19], [20], [42], [44]–[48], which support that late flowering is the ancestral *A. thaliana* state but a shift towards early flowering life cycle has recently occurred at the species level [2], [49].

In this study, we aim to determine the molecular basis of a novel *A. thaliana* flowering QTL named as *FAQ1*, which we identified in introgression lines developed by phenotypic selection from the Japanese accession Fukuyama (Fuk) and the reference strain Landsberg *erecta* (L*er*). Complementation in transgenic lines and directed mutagenesis demonstrated that a single amino acid substitution in the MADS-box gene *SHORT VEGETATIVE PHASE* (*SVP*) causes the early flowering of *FAQ1* allele present in Fuk accession. We further address the biogeography of *SVP* allelic variation showing that this is regionally structured because *FAQ1/SVP*-Fuk allele appeared confined to Asia and, most likely, it originated in Japan. In addition, we aim to quantify the extent of genetic interactions involving natural *SVP* alleles by developing and characterizing transgenic lines for Fuk and L*er SVP* alleles in five genetic backgrounds. These analyses show that *FAQ1/SVP* flowering effects vary from small to large depending on the genetic background, hence revealing the significant contribution of epistasis to the evolution of the flowering time variation mediated by *FAQ1/SVP*.

## Results

### *FAQ1* is a novel flowering QTL affecting the photoperiod response

**Figure 1:**
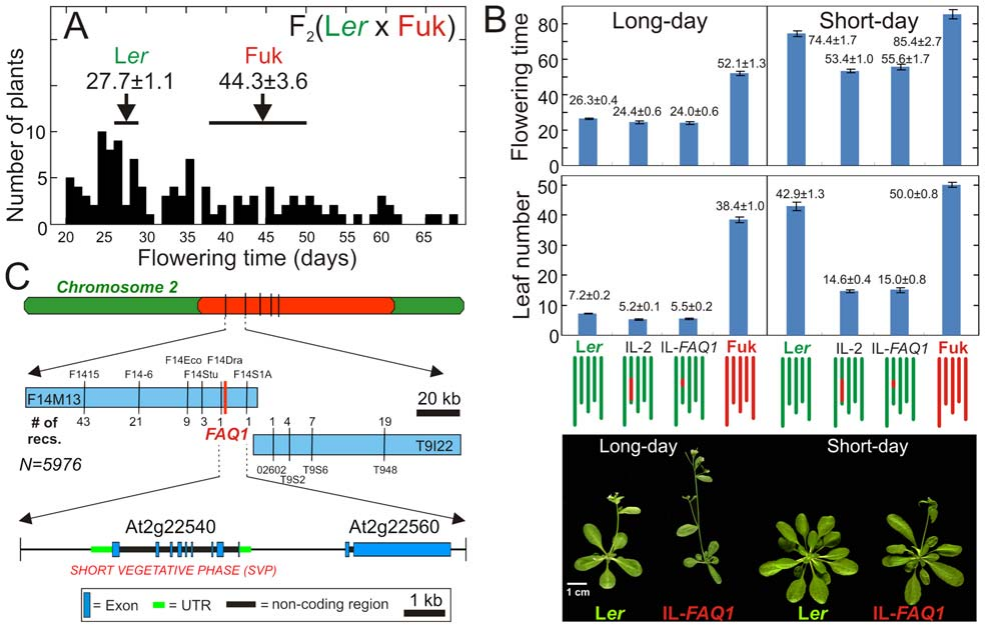
Identification, characterization, and mapping of *FAQ1*. A) Frequency distribution of flowering time in an F_2_ (L*er*×Fuk) population. Arrows and horizontal bars indicate the mean and range of variation of parental accessions. B) Flowering behaviour of ILs carrying *FAQ1*-Fuk alleles, grown under long-day and short-day photoperiods. Bars correspond to mean ± SE of 10–18 plants. Graphical genotypes are shown below the bars. In the lower panel, representative L*er* and IL-*FAQ1* plants photographed 24 days (for long-day) or 51 days (for short-day) after germination, are shown. C) Fine mapping of *FAQ1* showing the location and number of recombination events found in the 5976 gametes analysed along the BAC contig.

In order to uncover natural genetic variation for flowering initiation that is not detected by direct phenotypic comparisons of wild accessions, we quantified transgressive segregation in F_2_ populations derived from crosses between several accessions and the reference strain Landsberg *erecta* (L*er*). Using this approach we selected the genotype Fukuyama (Fuk) because 36% of the F_2_ individuals showed transgressive flowering times that duplicate the phenotypic variation observed between both parents (Figure 1A). To identify the loci responsible for this variation we developed introgression lines by phenotypic selection for flowering time during four backcross generations (see Materials and Methods). Two early flowering lines, IL-2 and IL-*FAQ1*, carrying single Fuk introgressions from chromosome 2 (of ∼9 and ∼2 Mb, respectively) in an otherwise L*er* genetic background, were characterized for their flowering behaviour (Figure 1B). On average, the two lines flowered two days earlier and with two leaves fewer than L*er* under long-day (LD) photoperiod. In contrast, under short-day (SD), both ILs flowered 21 days earlier and with 28 leaves less than the reference strain, which indicates that, similar to Fuk accession, these lines have a reduced response to photoperiod (Figure 1B). F_1_ hybrids derived from L*er* and the ILs showed towards-early intermediate flowering phenotypes suggesting incomplete dominance (Table S1). Thus, we identified a new large effect locus contributing to the natural variation for flowering initiation and its photoperiodic response, which was named as *Flowering Arabidopsis QTL1* (*FAQ1*).

### *SVP* is the gene underlying *FAQ1*

**Figure 2:**
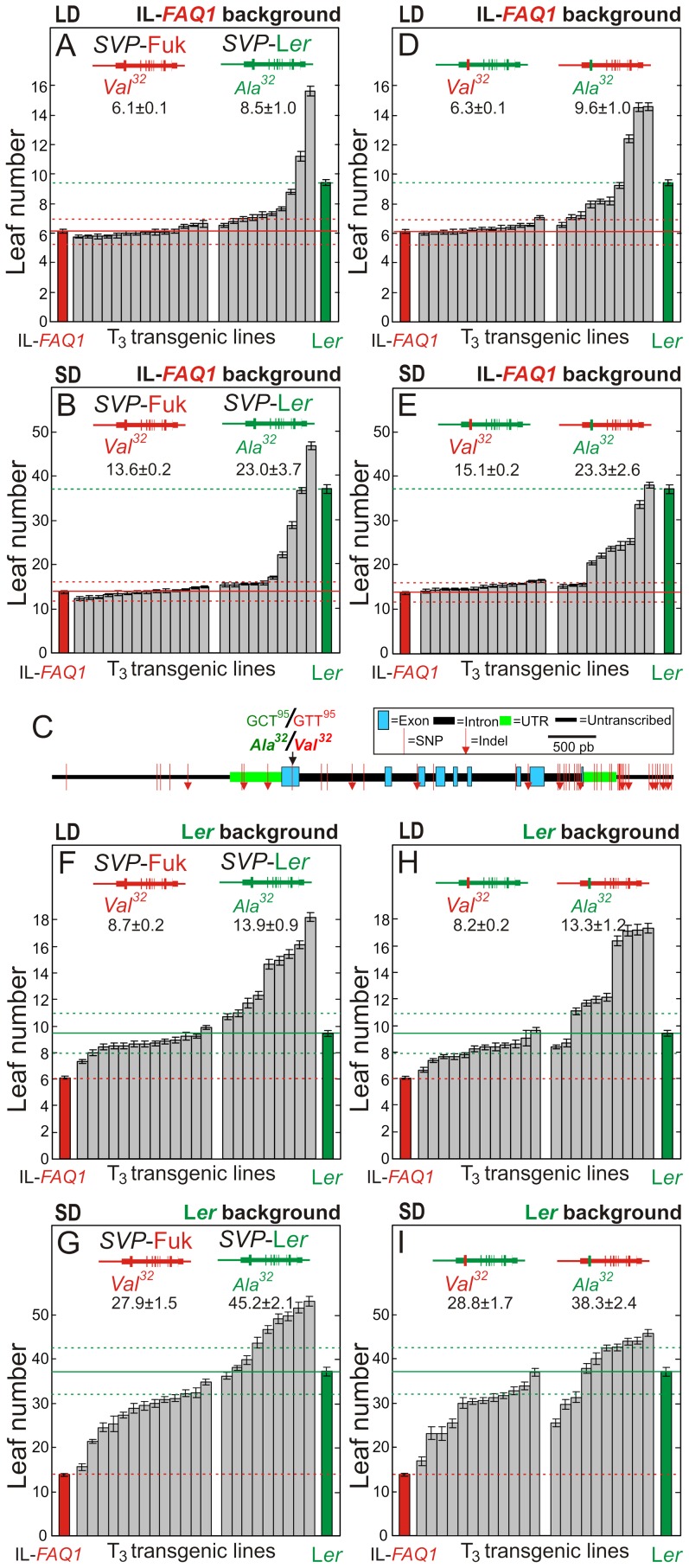
Flowering phenotypes of transgenic lines for parental and chimerical *SVP* alleles. Leaf number of independent homozygous T_3_ transgenic lines carrying parental (A, B, F and G) or chimerical (D, E, H and I) *SVP* genomic constructs in IL-*FAQ1* (A, B, D and E) or L*er* (F–I) genetic backgrounds. Lines were grown under long-day (LD) (A, D, F and H) or short-day (SD) (B, E, G, and I) photoperiods. C) Nucleotide polymorphisms found between *SVP* genomic sequences of L*er* and Fuk. Parental and chimerical *SVP* transgenes derived from Fuk (red colour) and L*er* (green colour) are depicted in the upper part of each panel. Bars are means ± SE of 10–15 plants per line. Mean ± SE of all lines carrying the same transgene are shown above the bars. Dashed lines delimit the 95% confidence intervals of the leaf number observed in untransformed IL-*FAQ1* (red colour) and L*er* (green colour) control lines, as established by Bonferroni tests.

Fine mapping using an F_2_ (L*er*×IL-2) population of 2988 individuals located *FAQ1* within a genomic interval of 11 kb where Col reference genome sequence contains only two open reading frames (Figure 1C). One of them, At2g22540, corresponded to the previously known flowering gene *SHORT VEGETATIVE PHASE* (*SVP*) encoding a MADS-box transcription factor [50]. To test if *SVP* might be *FAQ1*, we generated two *SVP* genomic constructs corresponding to L*er* and Fuk *SVP* alleles, and used them to transform plants of the early flowering line IL-*FAQ1* (Figure 2A and 2B). Homozygous transgenic lines carrying *SVP*-Fuk transgene did not differ in their flowering behaviour from IL-*FAQ1* indicating that this allele, in this genetic background, has no effect on flowering initiation. By contrast, most transgenic lines for *SVP*-Ler flowered significantly later than control plants, under SD and/or LD photoperiods (Figure 2A and 2B). Since *SVP*-L*er*, but not *SVP*-Fuk, transgenes largely complemented the early flowering and the reduced photoperiod response of IL-*FAQ1*, it was concluded that *SVP* underlies *FAQ1*.

### A single amino acid substitution is the *SVP*/*FAQ1* causal polymorphism

Sequencing of *SVP* in the parental accessions identified 50 single nucleotide polymorphisms (SNPs) and small indel polymorphisms differing between L*er* and Fuk (Figure 2C). Most polymorphisms were detected in non-coding genomic regions and only one non-synonymous SNP was found, which was located in the middle of the MADS domain. This mutation is predicted to change L*er* Ala^32^ to Fuk Val^32^, Ala^32^ appearing conserved in all SVP-like proteins (Figure S1). To evaluate the functional effect of this substitution we developed two additional chimerical *SVP* genomic constructs corresponding to L*er* and Fuk alleles where we replaced by directed mutagenesis Ala^32^ with Val^32^, and *viceversa*. In IL-*FAQ1* genetic background, homozygous transgenic lines carrying *SVP*-L*er*-Val^32^ transgene flowered similar to IL-*FAQ1* and did not differ from transgenic lines for *SVP*-Fuk allele (*P*>0.05; Figure 2D and 2E). However, most transgenic lines bearing *SVP*-Fuk-Ala^32^ transgenes flowered significantly later than control plants, under LD and SD photoperiod conditions. These results demonstrated that this single amino acid substitution strongly alters *SVP* function, Val^32^ from Fuk generating a *SVP* loss-of-function allele that displays no effect on flowering initiation, while L*er* Ala^32^ renders *SVP* functional and delays flowering initiation.

### *SVP* allelic interaction explains *FAQ1* incomplete complementation

Even though most IL-*FAQ1* transgenic lines carrying L*er* Ala^32^ in *SVP* transgene flowered later than IL-*FAQ1*, quantitative analysis of these lines showed that on average they flowered earlier than L*er* (Figure 2A and 2B). Therefore, *FAQ1* complementation with *SVP* transgenes was incomplete. To test if this was due to the existence of an additional gene linked to *SVP* that might contribute to *FAQ1*, or to an interaction between the transgenic and the endogenous copies of *SVP*, we used the four *SVP* genomic constructs to transform also L*er* plants (Figure 2F–2I). The four classes of L*er* transgenic lines showed the same overall flowering patterns observed in IL-*FAQ1* background. However, most transgenic lines carrying Fuk Val^32^ flowered earlier than L*er*, while most lines carrying L*er* Ala^32^ flowered significantly later than L*er* under SD and/or LD photoperiods (Figure 2F and 2G). The effect of *SVP* alleles was estimated in each background by comparing the transgenic lines carrying L*er* and Fuk transgenes (Table 1). Thus, *SVP* effect in L*er* background was significantly larger than in IL-*FAQ1* (*P*<0.05) and similar to *FAQ1* effect estimated by comparing L*er* and IL-*FAQ1* control lines. These results indicated that *SVP* accounts for most *FAQ1* effect but *SVP* transgenes interact with the genetic background. Since both backgrounds, L*er* and IL-*FAQ1*, differed only in the small introgression containing *SVP* gene, the *SVP* transgene most likely interact with the endogenous allele of *SVP*.

**Table 1:** *FAQ1/SVP* allelic effects on flowering initiation in different genetic backgrounds.

Genetic background	Endogenous *SVP* allele	Transgenes	# of Fuk/L*er* transgenic lines	Experiment	LD *FAQ1* effect	SD *FAQ1* effect
L*er* 1	L*er*, Fuk	no transgene	-	1	3.3	23.2
IL-*FAQ1*	Fuk	*SVP*-L*er*, *SVP*-Fuk	13/10	1	3.4	14.3
L*er*	L*er*	*SVP*-L*er*, *SVP*-Fuk	14/10	1	5.6	22.5
L*er* 1	L*er*, Fuk	no transgene	-	2	3.4	21
Pak-1	Fuk	*SVP*-L*er*, *SVP*-Fuk	15/10	2	0.9	1.8
Pak-3	Fuk	*SVP*-L*er*, *SVP*-Fuk	10/6	2	6.8	13.6
Fuk	Fuk	*SVP*-L*er*, *SVP*-Fuk	14/13	2	11.5	9.5
IL-*FAQ1*	Fuk	*SVP*-L*er*, *SVP*-Fuk	10/10	2	3.5	10.3
L*er*	L*er*	*SVP*-L*er*, *SVP*-Fuk	10/10	2	5.9	18

For each background is shown: the endogenous and transgenic *SVP* alleles analysed, the number of independent homozygous transgenic lines evaluated, and the average *FAQ1/SVP* allelic effects in long-day (LD) and short-day (SD) photoperiod. Allelic effects were estimated in two experiments as the mean difference between the leaf number of transgenic lines carrying *SVP* transgenes from L*er* and Fuk. Only transgenic lines differing significantly from the corresponding untransformed control were used for allelic effect estimates.

1 : The allelic effect of the original *FAQ1* locus (detected in L*er* and IL-*FAQ1* lines) was estimated as the leaf number difference between L*er* and IL-*FAQ1* untransformed plants.

### *SVP/FAQ1* flowering effects involve epistatic interactions

**Figure 3:**
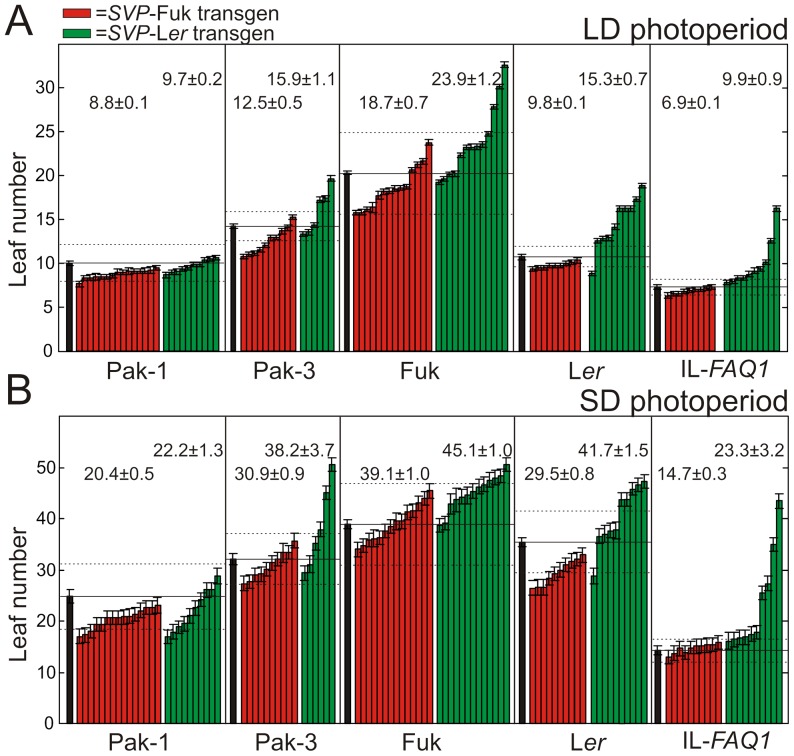
Flowering phenotypes of *SVP* transgenic lines developed in multiple genetic backgrounds. Leaf numbers of independent homozygous T_3_ transgenic lines carrying Fuk (red colour) or L*er* (green colour) *SVP* transgenes grown under long-day (LD) (A) or short-day (SD) (B) photoperiod. Genetic backgrounds are indicated in the horizontal axis. Bars are means ± SE of 10–15 plants per line. Mean ± SE of all lines carrying the same transgene and background are shown above the bars. Dashed lines delimit the 95% confidence intervals of the leaf numbers observed in the corresponding untransformed control lines as established by Bonferroni tests.

To further evaluate the genetic-background-dependency of *FAQ1/SVP* effect, we used the two *SVP* genomic constructs corresponding to L*er* and Fuk alleles to transform three additional accessions (Fuk, Pak-1 and Pak-3) carrying similar loss-of-function *FAQ1/SVP-*Fuk allele (see later). A total of 108 homozygous transgenic lines were selected in all five backgrounds and grown together under LD and SD photoperiods (Figure 3). The joint analysis of these lines showed strong additive effects of *SVP* transgenes and genetic backgrounds (*P*<0.001; Table S2). However, this quantitative analysis also detected significant *SVP* transgene by background interaction (*P*<0.01; Table S2) indicating that the allelic effect of *SVP* depends on the genetic background. This interaction was mainly determined by the small effect of *SVP* transgenes in Pak-1, since significant interactions were detected (*P*<0.05) in all pair comparisons of Pak-1 transgenic lines with the rest of backgrounds. As shown in Figure 3, in Pak-1, the two allelic classes of *SVP* transgenic lines differed weakly under both photoperiods (Table 1). In contrast, both classes of transgenic lines showed larger differences in the other backgrounds, the largest *SVP* allelic effect appearing in L*er* (Figure 3). Furthermore, the three-way interaction among *SVP* transgene, genetic background and photoperiod was significant (*P*<0.01; Table S2) evidencing that the effect of *SVP* on the flowering photoperiod response also depends on the genetic background. This is illustrated with the comparable *SVP* effect observed in Fuk, Pak-3 and IL-*FAQ1* lines when grown under SD, but not under LD photoperiod where Fuk lines displayed larger *SVP* allelic effect (Figure 3 and Table 1). Therefore, the differential behaviour of transgenic lines in backgrounds bearing the same endogenous *FAQ1/SVP* allele indicates that *SVP* transgenes interact with one or several genomic regions other than *SVP* locus, as well as with the photoperiodic environment.

### *SVP/FAQ1* loss-of-function allele shows a regional distribution in Asia

**Figure 4:**
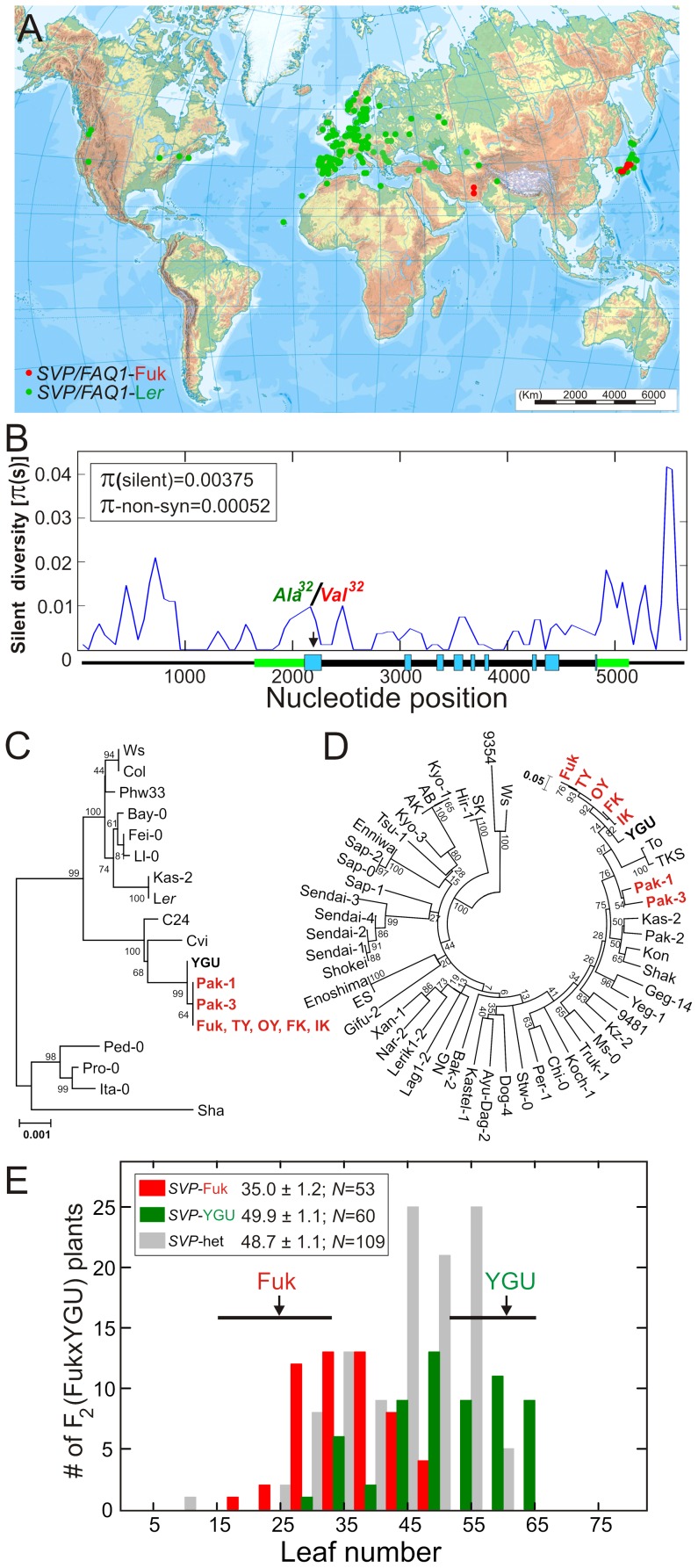
Geographic and genetic diversity patterns of natural *SVP* alleles. A) Geographic distribution of *SVP/FAQ1* causal polymorphism. B) Sliding window plot of nucleotide diversity along *SVP* region derived from 18 world-wide accessions. Nucleotide diversities in *SVP* coding region are shown inside the panel. C) N-J tree showing the genetic relationships among *SVP* sequences. D) N-J tree showing the genome-wide genetic relationships among 54 Asian accessions, as estimated from a set of 237 polymorphic SNPs. In C and D, accessions carrying Fuk allele for *SVP/FAQ1* causal polymorphism are shown in red color. E) Frequency distribution of leaf numbers in an F_2_ (Fuk×YGU) population. Average leaf number ± SE and sample size (*N*) of the three *SVP* genotypic classes, established based on Ala/Val^32^ CAPS marker, is given inside the panel.

Genotyping of a world-wide collection of 289 *A. thaliana* accessions with a CAPS marker specific for *SVP* causal polymorphism detected six additional accessions carrying Fuk Val^32^, two from Pakistan and four from Japan (Figure 4A). This showed that *SVP* causal polymorphism is geographically structured, Fuk loss-of-function allele appearing as rare at a global scale (<2.5% frequency) but common at a regional scale in Japan, where it displayed a frequency of ∼15%.

Sequencing analysis revealed that all seven accessions with Fuk Val^32^ carried the same *SVP* loss-of-function allele because they only differed in the length of a short AT-microsatellite located in the first intron. Further *SVP* sequencing in 18 accessions covering the world distribution (Figure 4B and 4C) showed an overall low nucleotide diversity in *SVP* coding region (π-silent = 0.0038), which increased up to average genome levels [51] only in the 5′ and 3′ flanking regions. Non-synonymous diversity was especially low because only the Ala^32^ to Val^32^ substitution was found, and no other polymorphism with obvious potential effect on *SVP* function was detected (Table S3). To determine the genetic relationships among accessions carrying *SVP* loss-of-function alleles we genotyped a sample of 54 Asian accessions for a set of 237 genome-wide SNPs (Figure 4D). The five Japanese accessions carrying Fuk Val^32^ were nearly identical with an average proportion of allelic differences (genetic distance) of 1.6%. However the two Pakistan genotypes carrying similar *SVP* allele differed substantially between them (9% genetic distance) and from Japanese accessions (average distance of 13.2%), although all these accessions were more related than other Asian genotypes.

### Functional allelic variation at *SVP/FAQ1* most likely originated in Japan

Sequence and genotypic analyses identified YGU as a Japanese genotype that is very close to the five Japanese accessions bearing Fuk Val^32^, for the overall genetic background (genetic distance of 5.6%) and for *SVP* haplotype (Figure 4C and 4D). However, YGU carried the active Ala^32^ *SVP* allele, the only other *SVP* nucleotidic difference corresponding to the length of the first intron microsatellite. Furthermore, YGU flowered significantly later than Fuk and the remaining Val^32^ accessions (Table S1), suggesting that *SVP* accounts for these flowering differences. This was strongly supported by co-segregation analysis in an F_2_ (Fuk×YGU) population grown under LD photoperiod, where *SVP* causal polymorphism explained 43% of the flowering phenotypic variance (Figure 4E). Thus, in this Fuk/YGU homogeneous genetic background, *SVP/FAQ1* displayed a large LD effect, in agreement with the behaviour of Fuk transgenic lines. Therefore, *SVP* loss-of-function allele was probably generated recently in Japan, and after outcrossing and recombination it expanded to Middle Asia.

## Discussion

### *FAQ1/SVP* sets MADS transcription factors as the main gene family accounting for natural flowering variation in *A. thaliana*

Despite the large number of flowering time QTL identified in *A. thaliana*, the molecular bases of only a dozen of them have been determined until now (see Introduction). In this work, we have isolated *FAQ1*, a new QTL identified as a large effect locus in a population highly trangressive for flowering initiation. Most previous studies have used permanent RIL populations or F_2_ families to detect and map QTL [9], [10], [20]. However, we identified this locus in a population of introgression lines developed by phenotypic selection in a homogeneous reference genetic background. Although the construction of such biological materials requires considerable time, they facilitated the later characterization, the fine mapping and the molecular isolation of *FAQ1*, showing the power of phenotype-based ILs as an alternative mapping resource to standard experimental populations.

We have demonstrated that the well-known regulator *SVP* encoding a MIKC-type MADS transcription factor [50], [52] contributes to the natural variation for flowering initiation in *A. thaliana*. It has been previously shown that *SVP* is a flowering repressor that affects the photoperiod response by negatively regulating several integrator genes such as *FT* and *SOC1* [53], [54]. *SVP* appears regulated by the circadian clock and by the autonomous, the thermosensory and the gibberellin pathways [53], [55], [56], which suggests that *SVP* is also a flowering pathway integrator. Network and protein interaction studies have further revealed that SVP is down-regulated by AP1 and interacts with AP1 and other floral MADS transcription factors like CAL and SEP3 [57]–[59] thereupon showing the close regulation between *SVP* and the flower identity genes. In addition, SVP binds to the promoters and regulates the expression of other transcriptional regulators including miR172 and several floral repressors of the AP2 family [60]. In this study we have proven that the natural amino acid substitution Ala^32^ to Val^32^, in the MADS domain, generates a *SVP* loss-of-function allele that cause early flowering, in agreement with the phenotypes described for artificial *svp* mutants [50], [53]. MADS domains are required for DNA binding but the Ala^32^, highly conserved among species, has been shown to participate also in MADS protein dimerization [61]. These functions suggest that SVP-Fuk-Val^32^ is likely unable to properly bind and repress *SOC1* and/or *FT* promoters, leading to the early flowering and reduced photoperiod sensitivity observed in Fuk accession. In addition, the specificity and uniqueness of this natural structural mutation suggest that most *SVP* structural modifications are likely deleterious and that SVP protein is essential for *A. thaliana* survival in nature.

Natural regulatory and structural polymorphisms in three additional MADS-box genes, *FLC*, *FLM* and *MAF2*, have been shown to affect flowering in *A. thaliana* [41]–[43], [62], [63]. In addition, a natural amino acid substitution in the MADS-box gene *AGL6* has been recently demonstrated to alter shoot branching in a flowering time dependent manner [64]. Moreover, an extensive *A. thaliana* genome-wide association study [32] has found S*VP* as associated with several flowering related traits, which suggests that additional *SVP* polymorphisms might affect flowering initiation. Hence, MIKC-type MADS transcription factors appear as the main class of genes accounting for the flowering natural variation in this species. Interestingly, another MADS-box gene homologous to *AP1* was found to contribute to the natural variation for vernalization flowering response in cereals [65]. Several studies have shown that *SVP*-like genes in different families of mono- and dicotyledonous plants display partially conserved functions in the photoperiod and vernalization flowering pathways [66]–[71] despite substantial copy number variation for *SVP*-like genes among species. Therefore, MADS transcription factors in general, and *SVP* in particular, appear as important candidate genes to explain the natural variation for flowering time or related traits also in plant families that are phylogenetically distant from *A. thaliana* [72].

### Genetic interactions determine the effects of natural *SVP* variation

Although *FAQ1/SVP* was detected as a large effect flowering QTL, quantitative analysis of transgenic lines shows that *FAQ1/SVP* effects vary from large to rather small as consequence of its genetic interactions. On the one hand, transgenic lines differing only in a small introgression indicate that *SVP* effect depends on the natural alleles in a genomic region located around *SVP*, which strongly suggests allelic interactions. This is best illustrated with the lack of flowering effects observed for *SVP*-Fuk-Val^32^ transgenes in the *SVP* loss-of-function background of IL-*FAQ1*, whereas these transgenes accelerated flowering in the near isogenic background of L*er*. Thus, the flowering repression of active *SVP*-L*er* alleles seems to be reduced by the presence of *SVP*-Fuk loss-of-function alleles. This result is in agreement with the incomplete dominance observed in hybrid plants derived from IL-*FAQ1* and L*er*, which cannot be explained simply by a *SVP* dosage effect [50]. Since the function of MADS transcription factors involves homo- and hetero-dimers [57], [58] it can be speculated that in plants bearing both natural *SVP* alleles, protein complexes containing SVP-Val^32^, directly or indirectly, reduce the overall SVP transcriptional repressing capacity. On the other hand, transgenic lines in different genetic backgrounds carrying the same endogenous loss-of-function *SVP* allele show that *SVP* effects depend on the natural alleles in other genomic region(s), which implies significant *SVP* epistatic interactions. Interestingly, SVP interacts physically with several MADS transcription factors like FLC, AP1, SOC1 and AGL6 [53], [56], [57]. This suggests that the functional basis of the observed *SVP* genetic interaction is the physical interaction between SVP protein and other MADS transcription factors involved in multiple complexes. Such interactions could also account for the genetic-background-dependency observed for the incomplete dominance of *SVP* alleles because, in contrast to the behavior in F_1_(L*er*×IL-*FAQ1*) plants, *SVP*-Fuk allele behaved nearly as recessive in the F_2_(Fuk×YGU) population (Figure 4E).

All flowering QTL isolated so far correspond to large effect alleles [9], [10], which has hampered our understanding of the molecular mechanisms involved in the natural variation for flowering initiation mediated by small effect QTL [73]. The genetic-background-dependency of *FAQ1/SVP* shows that QTL that are primarily detected as large effect loci may have varying effects owing to genetic interactions. Thus, epistasis appears as an important component of QTL effect estimation, which is often neglected in Fisher's views of natural quantitative variation that assume the existence of series of alleles with different additive effects [39], [74]. This result brings the possibility that some of the natural flowering alleles previously isolated might also underlie flowering QTL detected with small effect, a hypothesis whose testing requires the analysis of genetic interactions in multiple backgrounds, as shown here for *FAQ1/SVP*. In particular, natural variants of gene families that participate in multimer protein complexes, such as the MADS genes [57], are expected to show significant genetic interactions [39], as described for numerous artificial mutant alleles of these genes including *SVP*, *FLM* and *FLC* [55], [58], [75]–[78]. This view is also supported by the recent identification of a natural allele of *AGL6* that affects axillary bud formation in an epistatic manner [64]. It can then be speculated that the natural *SVP* interacting partners are any of the MADS genes *FLM*, *FLC*, *MAF2* or *AGL6*, as supported by their segregation in nature and their participation in *SVP* genetic and physical interactions, although we cannot discard other genes. Thus, our study shows the usefulness of quantitative analyses of transgenic lines in multiple genetic backgrounds as a general approach to uncover any order (di- and higher-order) genetic interactions with specific natural alleles. Nevertheless, given the significant variation found among transformants, this method demands the generation of large numbers of independent transgenic lines.

### *SVP* natural allelic variation is probably involved in *A. thaliana* adaptation

Most *A. thaliana* alleles that have been functionally demonstrated as contributing to the natural variation for flowering initiation are alleles found in a unique accession, which hampers inferences about their role in plant adaptation [9]. By contrast, the early flowering *SVP*-Fuk allele appears as a recent allele likely originated in Japan and distributed in Asia. Several arguments support that this genetic variant is involved in adaptation. First, its moderate frequency in Asia, in accessions that belong to genetically differentiated clades, indicates that this is not a deleterious allele to be purged from a unique local population. Phenotypic analysis of *FAQ1* ILs did not detect any other obvious developmental alteration, further supporting flowering specificity and absence of negative pleiotropic effects of *SVP*-Fuk allele. Second, *SVP*-Val^32^ is the only detected amino acid substitution that has been maintained in nature at high regional frequency, whereas low silent and non-synonymous nucleotide diversities suggest that *SVP* is under purifying selection. Third, its early flowering phenotype is in agreement with the strong recent directional selection favouring earliness that has been described at the species level [2], [49]. The significant *SVP* flowering effect in Fuk/YGU genetic background, in which most likely *SVP*-Fuk allele was originated, supports that natural selection could act through the *SVP*-Fuk earliness. Thus, in addition to *FRI*, *FLC* and *MAF2* genes harbouring several frequent loss-of-function mutations [13], [15], [19], [41], [46]–[48] *SVP* represents another flowering repressor (or vegetative growth promoter) that might be under natural selection for early flowering, in agreement with previous predictions [2]. The limited regional distribution of *SVP*-Fuk is probably determined by its short demographical history in a non-native region that has been recently colonized [11]. However, *SVP* might be involved in adaptation to particular Asian local environments. The presence of this allele in a set of genetically related accessions suggests that such potential adaptive effect of *SVP*-Fuk depends on the genetic background, as supported by the genetic interactions described for *SVP* flowering effect. Conclusive demonstration of *SVP* contribution to adaptation awaits the analysis of the currently unknown environmental conditions where natural *SVP* alleles have evolved, as recently reported for other flowering genes in more extensively sampled and documented geographic regions [15], [27].

## Materials and Methods

### Plant materials

The laboratory strain Landsberg *erecta* (L*er*) and the wild accession Fuk, obtained from Sendai Stock centre (JW116; http://www.brc.riken.jp/lab/epd/Eng/catalog/seed.shtml) and originally collected around Fukuyama (Japan), were used as parental lines to develop a population of 31 introgression lines carrying Fuk genomic segments in L*er* background. ILs were developed by phenotypic selection for early flowering time during four backcross generations, each backcross being followed by a selfing generation. Briefly, the four earliest plants of an F_2_ (L*er*×Fuk) population of 120 plants were backcrossed to L*er* to obtain four independent families. A single early plant was selected per family in each of the following selfing and backcross generations. After four backcrosses, 7–8 individual sister plants per family (a total of 31 ILs) were thoroughly genotyped with 100 AFLP, microsatellite and indel polymorphic markers previously described [26], [79], [80].

IL-2 carrying a single introgression fragment of ∼9 Mb in chromosome 2 was crossed to L*er* to obtain a *FAQ1* F_2_ mapping population. *FAQ1* was fine mapped by genotyping 2988 F_2_ plants with 24 CAPS and indel markers developed from different sources. IL-*FAQ1*, carrying an introgression of ∼2 Mb between physical positions 7.6 and 9.6, was derived from the mapping population.

A world-wide collection of 189 accessions (Table S4) and a collection of 100 Iberian wild genotypes [81] were analysed for flowering behaviour, for *SVP* sequence, and/or for *SVP* causal polymorphism.

### Growth conditions and measurements of flowering initiation

Plants were grown in pots with soil and vermiculite at 3∶1 proportion in an air-conditioned greenhouse at 21°C, supplemented with additional light to provide long-day photoperiod (16 h light∶8 h darkness). For short-day photoperiod evaluations (8 h light∶16 h darkness) plants were grown in a growth chamber illuminated with cool-white fluorescent lamps.

Flowering initiation was measured as leaf number and flowering time. Leaf number was calculated as the total number of rosette and cauline leaves in the main inflorescence. Flowering time was estimated as the number of days from the planting date until the opening of the first flower.

### *SVP* sequences, constructs, and transgenic lines

A *SVP* genomic fragment of 6.5 kb, including 3.2, 2.4 and 0.9 kb of the coding, the 5′ and the 3′ regions, respectively, were sequenced in L*er* and Fuk. A 5.6 kb *SVP* segment was sequenced in other 15 accessions (Table S4). Nine to 12 overlapping fragments of 0.8–1.3 kb were PCR amplified (Table S5) and products were sequenced using an ABI PRISM 3700 DNA analyzer. DNA sequences were aligned using DNASTAR v.8.0 (Lasergene) and alignments were inspected and edited by hand with GENEDOC [82]. Nucleotide diversity, recombination and linkage disequilibrium were estimated with DnaSP v.5 [83]. GenBank accession numbers of DNA sequences generated in this work are JX863084–JX863100.

The two 6.5 kb *SVP* genomic fragments from L*er* and Fuk were cloned in pCAMBIA2300 binary vector (CAMBIA, Canberra, Australia) by standard molecular biology techniques. Briefly, three successive *SVP* segments were PCR amplified and cloned in appropriate cloning sites, and subsequently fused in the right orientation (Table S5). Two additional *SVP* chimerical constructs were derived by reciprocally replacing the SNP causing Ala^32^ to Val^32^ substitution. For that, site-directed mutagenesis of this SNP was performed by PCR using the spliced overlap extension method as described by Hepworth et al. [84]. Primers containing the nucleotide to be replaced are shown in Table S5. The two PCR products of each accession were purified, mixed, and subjected to 12 PCR cycles to allow extension of heteroduplexes formed between the overlapping sequences. Extended heteroduplexes were then amplified with oligonucleotides *SVP-Bam*HI-F and *SVP*-*Bam*HI-R, digested with *Bam*HI and *Xba*I, gel purified, and used to replace the fragment *Bam*HI/*Xba*I in L*er* and Fuk SVP constructs. All PCR amplifications were performed using high fidelity *Pfu* polymerase (Promega, Wisconsin, USA) and constructs were verified by sequencing.

*SVP* genomic constructs were transferred by electroporation to AGL0 *A. tumefaciens* strain [85] and plants of *A. thaliana* were transformed by the floral dip method [86]. T_1_ transformants were screened by kanamycin resistance and lines carrying single insertions were selected based on resistance segregation in T_2_ families. Ten to 14 independent homozygous T_3_ lines were selected for each construct and genetic background, their transgene and endogenous *SVP* alleles being verified by PCR (Table S5) previous to phenotypic analyses. Phenotypic differences among transgenic lines were tested statistically with general linear models using SPSS v 19.0.

### SNP genotyping and clustering analyses

Collections of accessions were genotyped using a CAPS marker specifically developed for *SVP* causal polymorphism (Table S5). Accessions from Asia were further genotyped for a genome-wide set of 320 SNPs selected from different sources, as previously described [81], [87]. A total of 237 SNPs were polymorphic and were used for genetic distance and clustering analyses, their average missing data being 4.8%. Neighbor-Joining (N-J) trees were constructed with MEGA5 [88] using 10000 bootstraps to calculate percent support for each branch node.

## Supporting Information

Figure S1 Sequence comparison of MADS domains of SVP and MADS proteins from different species. The alignment includes 30 SVP proteins from 22 plant species and 10 MADS related proteins from six species. *FAQ1* causal polymorphism between L*er* and Fuk accessions (Ala^32^ to Val^32^) is indicated, and the conserved L*er-*Ala^32^ is highlighted. Genbank accession numbers of the protein sequences included are as follow: SVP from *Arabidopsis thaliana* (ABU95408.1); AGL24 from *A. thaliana* (NP_194185.1); SVP from *A. lyrata* (EFH54881); SVP from *Brassica rapa* (ABG24233.1); SVP from *B. napus* (AFG73587.1); SVP from *B. juncea* (AFG73588.1); SVP from *Medicago truncatula* (XP_003613054.1); SVP from *Pisum sativum* (AAX47170.1); SVP-like from *Glycine max* (ABY78023.1); JOINTLESS from *Solanum lycopersicum* (AAG09811.1); SVP-like 1 from *S. tuberosum* (AAB94006.1); SVP-like 2 from *S. tuberosum* (AAV65507.1); JOINTLESS from *Malus domestica* (ABD66219.2); SVP1 from *Actinidia chinensis* (AFA37967.1); SVP2 from *A. chinensis* (AFA37968.1); SVP3 *A. chinensis* (AFA37969.1); SVP4 from *A. chinensis* (AFA37970.1); SVP-like from *Citrus trifoliata* (ACJ09170.1); SVP-like 1 from *Vitis vinifera* (XP_002269295.1); SVP-like 2 from *V. vinifera* (AFC96914.1); SVP-like 3 from *V. vinifera* (XP_002285687.1); SVP-like from *Eucalyptus occidentalis* (AAP40641.1); SVP-like from *Coffea arabica* (ADU56833.1); SVP-like from *Marchantia polymorpha* (ADB81895.1); SVP-like 1 from *Ipomoea batatas* (BAC15562.1); SVP-like 2 from *I. batatas* (BAC15561.1); SVP-like from *Oryza sativa* (Q9XJ66.1); SVP-like 1 from *Hordeum vulgare* (CAB97349.1); SVP-like 2 from *H. vulgare* (DQ201168.1); SVP-like from *Zea mays* (NP_001105148.1|); SVP-like from *Brachypodium distachyon* (XP_003581663.1); SVP-like from *Physcomitrella patens* (XP_001779871.1); AGAMOUS from *A. thaliana* (AEE84111.1); APETALA 3 from *A. thaliana* (P35632.1); SRF from *Homo sapiens* (NP_003122.1); MSEF2 from *H. sapiens* (NP_002388.2); MEF2 from *Xenopus laevis* (NP_001089962.1); SRF from *Drosophila melanogaster* (NP_726438.1); MEF2 from *D. melanogaster* (NP_995789.1); Mcm1p from *Saccharomyces cerevisiae* (CAA88409.1)
(TIF)

Table S1 Flowering behaviour of genotypes with different natural *SVP* alleles.
(XLS)

Table S2 General linear model testing the effects of *SVP* transgenes, the genetic background and the photoperiod in transgenic lines.
(XLS)

Table S3 *SVP* nucleotide diversity.
(XLS)

Table S4 *A. thaliana* natural accessions analyzed for *SVP* sequence and causal polymorphism.
(XLS)

Table S5 Oligonucleotides used for *SVP* sequencing, accession genotyping, cloning and verification of transgenic lines.
(XLS)
