# Supplementary figures and images for: The Flowering Repressor SVP Underlies a Novel Arabidopsis thaliana QTL Interacting with the Genetic Background
Source: PLoS Genet. 2013 Jan 31;9(1):e1003289. doi: 10.1371/journal.pgen.1003289 (PMC3561112; doi:10.1371/journal.pgen.1003289)

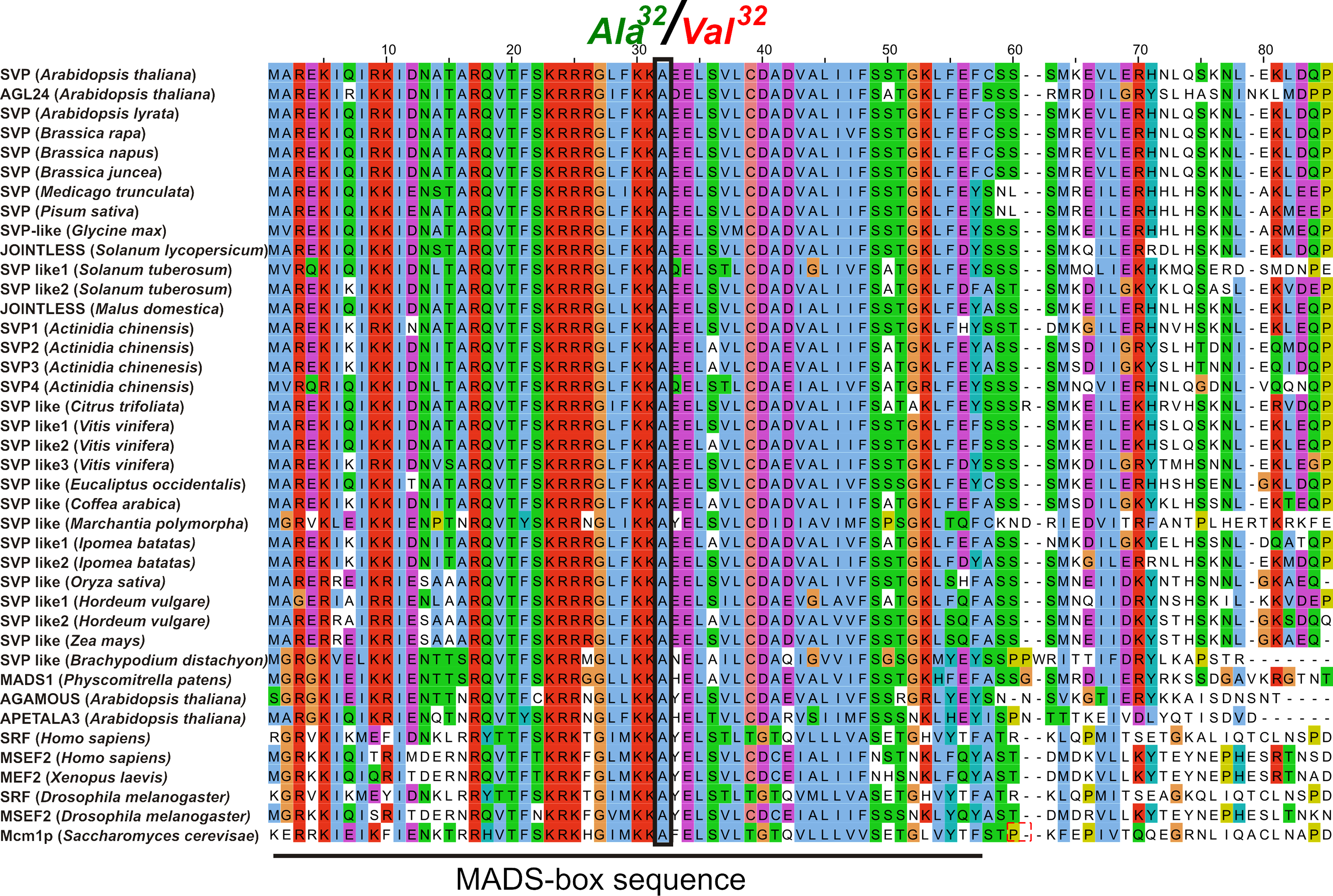

Supplement: Figure S1 — Sequence comparison of MADS domains of SVP and MADS proteins from different species. The alignment includes 30 SVP proteins from 22 plant species and 10 MADS related proteins from six species. FAQ1 causal polymorphism between Ler and Fuk accessions (Ala32 to Val32) is indicated, and the conserved Ler-Ala32 is highlighted. Genbank accession numbers of the protein sequences included are as follow: SVP from Arabidopsis thaliana (ABU95408.1); AGL24 from A. thaliana (NP_194185.1); SVP from A. lyrata (EFH54881); SVP from Brassica rapa (ABG24233.1); SVP from B. napus (AFG73587.1); SVP from B. juncea (AFG73588.1); SVP from Medicago truncatula (XP_003613054.1); SVP from Pisum sativum (AAX47170.1); SVP-like from Glycine max (ABY78023.1); JOINTLESS from Solanum lycopersicum (AAG09811.1); SVP-like 1 from S. tuberosum (AAB94006.1); SVP-like 2 from S. tuberosum (AAV65507.1); JOINTLESS from Malus domestica (ABD66219.2); SVP1 from Actinidia chinensis (AFA37967.1); SVP2 from A. chinensis (AFA37968.1); SVP3 A. chinensis (AFA37969.1); SVP4 from A. chinensis (AFA37970.1); SVP-like from Citrus trifoliata (ACJ09170.1); SVP-like 1 from Vitis vinifera (XP_002269295.1); SVP-like 2 from V. vinifera (AFC96914.1); SVP-like 3 from V. vinifera (XP_002285687.1); SVP-like from Eucalyptus occidentalis (AAP40641.1); SVP-like from Coffea arabica (ADU56833.1); SVP-like from Marchantia polymorpha (ADB81895.1); SVP-like 1 from Ipomoea batatas (BAC15562.1); SVP-like 2 from I. batatas (BAC15561.1); SVP-like from Oryza sativa (Q9XJ66.1); SVP-like 1 from Hordeum vulgare (CAB97349.1); SVP-like 2 from H. vulgare (DQ201168.1); SVP-like from Zea mays (NP_001105148.1|); SVP-like from Brachypodium distachyon (XP_003581663.1); SVP-like from Physcomitrella patens (XP_001779871.1); AGAMOUS from A. thaliana (AEE84111.1); APETALA 3 from A. thaliana (P35632.1); SRF from Homo sapiens (NP_003122.1); MSEF2 from H. sapiens (NP_002388.2); MEF2 from Xenopus laevis (NP_001089962.1); SRF from Drosophila melanogaster (NP_726438 [file pgen.1003289.s001.tif]
